# The primary cilium as a compartmentalized signaling hub in tissue immunity and homeostasis 

**DOI:** 10.3389/fcell.2026.1875556

**Published:** 2026-07-06

**Authors:** Anqi Zhang, Changjun Huo, Ting Song

**Affiliations:** Center for Cell Structure and Function, College of Life Sciences, Shandong Normal University, Jinan, China

**Keywords:** Cilia, ciliary signaling, ciliopathy, immune homeostasis, mucosal immunity

## Abstract

Cilia are evolutionarily conserved microtubule-based organelles that function as sensory and motile hubs central to mucosal immunity and systemic immune homeostasis. Motile cilia drive mucociliary clearance as a primary physical defense; meanwhile primary cilia function as signaling scaffolds that regulate immune receptor dynamics including PD-L1 trafficking. These organelles also modulate inflammatory responses through pathways such as cGAS-STING. Disruptions in ciliary assembly, intraflagellar transport, or signaling drive pathological immune dysregulation, contributing to viral infections, chronic inflammatory diseases, and ciliopathy-related immune dysfunction. This review summarizes core ciliary signaling pathways and their roles in host defense and immune homeostasis, integrating recent advances linking ciliary defects to diverse immune-related pathologies. We discuss current limitations in understanding ciliary immunobiology and propose key directions for future research aimed at deepening mechanistic insights and facilitating clinical translation.

## 1 Introduction

**FIGURE 1:**
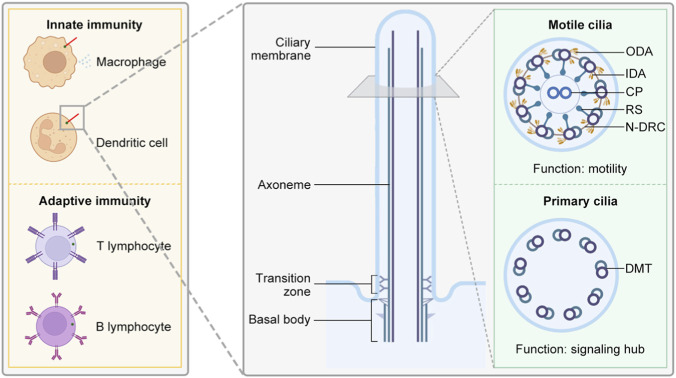
Structural organization and functional diversity of motile and primary cilia in immune regulation. (Left) Schematic representation of primary cilia or ciliary machinery components (indicated by green dots/red lines) within innate immune cells (macrophages and dendritic cells) and adaptive immune cells (T and B lymphocytes). While some immune cells possess distinct primary cilia, others repurpose ciliary trafficking components to orchestrate polarized signaling domains. (Right) Schematic representation of the general ciliary architecture, illustrating the ciliary membrane, axoneme, transition zone, and basal body. Transverse sections highlight key ultrastructural distinctions: motile cilia exhibit a “9 + 2” microtubule arrangement equipped with outer dynein arms (ODA), inner dynein arms (IDA), a central pair (CP), radial spokes (RS), and the nexin-dynein regulatory complex (N-DRC) to drive motility. In contrast, primary cilia possess a “9 + 0” architecture lacking the central pair and dynein arms, instead featuring nine outer doublets (DMT) to function as specialized signaling hubs.

Cilia are microtubule-based organelles protruding from the eukaryotic cell surface that serve as critical determinants of cellular function (Sakai et al., 2025; Zhu, 2025). While motile cilia primarily drive fluid transport in specialized tissues like the airway epithelium (Lyu et al., 2024; Song et al., 2025; Chen et al., 2026), primary (non-motile) cilia function as specialized evolutionary “cellular antennae” and distinct signaling compartments (Wang D. et al., 2025; Song et al., 2026). Defined by a “9 + 0” axonemal architecture, the primary cilium establishes a unique, segregated ciliary membrane microenvironment rich in specific receptors and ion channels (Reiter and Leroux, 2017). This compartmentalization, sustained by the regulated intraflagellar transport (IFT) machinery, allows the organelle to concentrate and process extracellular mechanical and chemical stimuli independently from the bulk cytoplasm, acting as a high-fidelity signaling scaffold (Lacey and Pigino, 2025; Klena and Pigino, 2022) (Figure 1).

Efficient immune surveillance and host defense require immune cells to rapidly interpret diverse extracellular signals while maintaining strict activation thresholds (Nguyen and Soulika, 2019; Jiang et al., 2025; Nahrendorf et al., 2025). However, traditional models based on stochastic surface receptor interactions often fail to explain how immune cells overcome spatial signaling constraints—such as the need to rapidly polarize receptors and segregate downstream cascades in a crowded tissue microenvironment (Shanker et al., 2015; Signa et al., 2022). For instance, innate immune cells (e.g., macrophages and dendritic cells) must precisely localize pattern recognition receptors (PRRs) to sense pathogen-associated molecular patterns (PAMPs) without triggering chronic systemic inflammation (Remick et al., 2023; Carroll et al., 2024; Kawai et al., 2024). Similarly, adaptive T and B lymphocytes rely on hyper-polarized membrane domains to achieve exquisite antigen sensitivity and orchestrate coordinated intercellular crosstalk (Rydyznski Moderbacher et al., 2020; Linge et al., 2022; Rappazzo et al., 2023). Accumulating evidence suggests that the primary cilium, or its molecular counterparts, provides the precise spatial and mechanistic platform required to resolve these signaling constraints.

Crucially, the presence and functional manifestation of this ciliary machinery vary distinctively across different immune cell lineages. While certain differentiated immune cells, such as specific subsets of macrophages and dendritic cells, utilize classical primary cilia to sense microenvironmental cues (Sutton et al., 2024; Toriyama et al., 2023), others like T cells lack a stable primary cilium but repurpose the evolutionarily conserved IFT machinery and centrosomal components to assemble the immunological synapse-a functional analogue of the ciliary signaling compartment (Gal et al., 2017; Finetti et al., 2011) (Figure 1). Within this framework, the primary cilium and its molecular remnants emerge as a sophisticated signaling hub that dictates immune cell fate, from activation thresholds to differentiation trajectories (Cassioli and Baldari, 2019; Anvarian et al., 2019). Accordingly, elucidating the role of this evolutionarily conserved organelle in the recognition and modulation of immune signaling represents a burgeoning interdisciplinary frontier at the intersection of cell biology and immunology.

## 2 Ciliary-mediated immune defense mechanisms under physiological conditions

To understand the ciliary-immune interface, it is essential to first delineate the organelle’s contributions under steady-state physiological conditions.

### 2.1 Ciliary force: physical barrier and pathogen clearance

The respiratory system’s primary role in gas exchange renders it inherently vulnerable to pathogens, necessitating a robust multi-layered defense to maintain mucosal integrity (Mannion et al., 2023; Yuan J. et al., 2025). At this interface, the host establishes a coordinated defense system by integrating physical and cellular strategies to obstruct microbial entry (Crystal et al., 2008; Ganesan et al., 2013). This barrier comprises the airway surface liquid replete with antimicrobial peptides and neutralizing immunoglobulins and a specialized epithelial cell layer (LeMessurier et al., 2020). While intercellular tight junctions provide a static seal to prevent debris from traversing into the internal environment, motile cilia function as the kinetic arm of innate immunity, driving continuous hydrodynamic removal and shaping boundary-layer dynamics to prevent pathogen colonization (Roy et al., 2014; Myszor and Gudmundsson, 2023).

**FIGURE 2:**
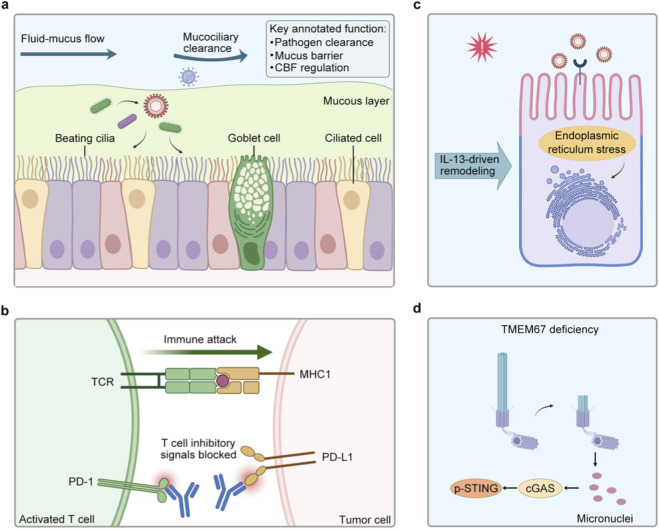
Multifaceted immune functions of cilia under homeostasis and pathological settings. **(a)** Under homeostatic conditions, motile cilia on the mucosal epithelium drive directional fluid-mucus transport to facilitate mucociliary clearance, the frontline innate defense against inhaled pathogens. Goblet cells secrete mucins to entrap microbes, while coordinated ciliary beating provides the mechanical force necessary to expel the mucus layer. Key physiological hallmarks include efficient pathogen clearance, maintenance of the mucus barrier, and the dynamic regulation of ciliary beat frequency (CBF). **(b)** Ciliary regulation of surface PD-L1 expression fine-tunes T-cell activation thresholds; blockade of the PD-1/PD-L1 pathway unleashes TCR-mediated immune attacks against tumor cells. **(c)** In type 2-high (T2H) asthma, IL-13 drives widespread epithelial remodeling, disrupting ciliary architecture and triggering endoplasmic reticulum (ER) stress in ciliated cells. **(d)** TMEM67 deficiency-induced ciliary defects trigger micronucleus formation, leading to cytosolic self-DNA sensing by cGAS and subsequent activation of the p-STING pathway.

Multiciliated cells (MCCs) generate unidirectional fluid propulsion through coordinated, metachronal beating, which drives the mucociliary escalator to expel inhaled pathogens and particulates (Crotta et al., 2023; Yuan L. et al., 2025; Liu et al., 2020). The efficacy of this defense is governed by the precise calibration of ciliary beating frequency (CBF) and the rheological properties of the mucus layer (Donoghue et al., 2023; Saito et al., 2023) (Figure 2a). Crucially, this mechanical system is dynamically tuned by immune mediators. Purinergic signaling such as ATP elevates CBF to accelerate clearance during acute infection (Sekiya et al., 2024; Kobayashi et al., 2024; Kamiya et al., 2020). Beyond horizontal transport, computational modeling underscores that this metachronal coordination is optimized to maximize fluid transport efficiency at the epithelial boundary layer. By generating coordinated shear force, ciliary arrays create a dynamic fluidic sweep that counteracts pathogen sedimentation, thereby minimizing microbial contact with the underlying epithelium (Causa et al., 2025).

This hydrodynamic clearance is further reinforced by resident leukocytes, which maintain airway homeostasis through constant surveillance and cytokine-mediated signaling (Lamichhane and Samarasinghe, 2019). However, highly adapted pathogens, such as influenza virus and *Streptococcus pneumoniae*, have evolved mechanisms to breach these defenses, preying on the respiratory tract for replication and causing significant host tissue damage (Monticelli et al., 2011; Wu et al., 2020; Earnhardt et al., 2023; Liu et al., 2024). Consequently, the ciliary array operates as a dynamic fluidic engine that synergizes with biochemical and structural defenses to safeguard host tissues.

### 2.2 Ciliary signaling: chemical defense and immune homeostasis

Beyond mechanical clearance, cilia orchestrate a sophisticated molecular defense network that integrates extracellular chemical cues with host cellular homeostasis. In the airway, the overlying mucus layer functions not only as a physical entrapment gel but also as a biochemical microenvironment that interfaces with ciliary sensory pathways (Ermund et al., 2021; Chatterjee et al., 2023; Tanaka et al., 2021). Recent advances have redefined cilia as specialized signaling hubs that integrate immune checkpoint regulation with tissue homeostasis (Agborbesong and Li, 2024; Bustos-Moran et al., 2017).

A burgeoning frontier in this field is the bidirectional crosstalk between primary cilia and immune checkpoint molecules, which serves as a physiological rheostat for immune tolerance. Cilia decorate and fine-tune the surface expression of programmed death-ligand 1 (PD-L1), thereby modulating T-cell activation thresholds and restricting aberrant autoinflammation (Agborbesong and Li, 2024; Tian et al., 2024; Ro et al., 2025) (Figure 2b). Conversely, PD-L1 functions as a molecular regulator for ciliogenesis through its precise trafficking and localization to the centrosome and Golgi apparatus. At these compartments, PD-L1 is strictly required for the functional recruitment of essential ciliary trafficking components, including the small GTPase Rab8a and the BBSome component BBS5 (Agborbesong and Li, 2024; Yu et al., 2023). This reciprocal interaction indicates that the primary cilium is not merely a passive docking platform for immune checkpoints, but an active regulatory hub governing PD-L1 biosynthesis and intracellular vesicular trafficking to maintain baseline immune quiescence.

Moving past surface-level signaling, cilia function as central gatekeepers of tissue homeostasis by orchestrating both secretory defense and structural integrity. In the respiratory tract, ciliated cells contribute to the immunoregulatory secretome by constitutively secreting IL-17D to restrict the recruitment of pathogenic macrophages, a secretory function intimately linked to the maintenance of healthy ciliated cell identity. This process is often disrupted in Type 2-high asthma where IL-13-driven remodeling leads to ciliary ER stress and the secretion of an innate immunity-depleted proteome (Yuan L. et al., 2025; Jackson et al., 2020) (Figure 2c). Distinct from this cell-intrinsic secretome, the extracellular barrier also requires proper mucin composition, where MUC5B (but not MUC5AC) acts as a critical external matrix component to maintain macrophage-mediated homeostasis and prevent chronic infection (Roy et al., 2014).

Complementing this mucosal defense, primary cilia also safeguard the internal cytoskeleton–nuclear envelope axis, particularly in mesenchymal and stromal compartments. For instance, in endometrial stromal cells, ciliary ablation induced by TMEM67 deficiency triggers a RhoA-dependent actomyosin contractile response that exerts pathological mechanical stress on the nucleus, leading to nuclear rupture and micronucleus formation. The subsequent collapse of these micronuclei releases double-stranded self-DNA into the cytosol, which is recognized by the cyclic GMP-AMP synthase (cGAS) sensor, thereby igniting the STING-dependent interferon pathway. This ectopic activation drives chronic sterile inflammation and impairs tissue-specific homeostatic processes such as decidualization (Li B. et al., 2025) (Figure 2d). Collectively, these findings position the cilium as an organelle-level integrator that bridges extracellular sensory perception with the maintenance of systemic immunological stability.

## 3 Ciliary dysfunction and immune dysregulation under pathological conditions

In contrast to its homeostatic roles under physiological conditions, structural and functional ciliary abnormalities serve as core driven mechanisms that precipitate pathological immune dysregulation across a range of diseases including acute viral infections, chronic inflammatory disorders and complex genetic syndromes. The associated cilia-related molecules and their immunological functions are summarized in Table 1.

**TABLE 1:** Cilia-related molecules in immune dysregulation.

Molecule	Mechanism	Associated disease
ACE2	Ciliary tip binding; facilitates viral entry	SARS-CoV-2 infection (Delta variant)
IL-17D	Homeostatic brake; limits allergic inflammation	Allergic airway hyperresponsiveness
IL-13	Goblet cell metaplasia; inhibits ciliation	Severe asthma/Allergic rhinitis
CCDC39	IDA assembly; triggers IL-6/IL-8 secretion	PCD (Severe inflammation)
DNAH5	ODA component; moderate inflammatory profile	PCD (Moderate inflammation)
DNAH11	ODA component; mild inflammatory profile	PCD (Mild inflammation)
CFTR	Airway hydration; NE-induced endocytosis	PCD/CF-related airway disease
CCL3	Stress-induced neutrophil chemoattractant	Nanoplastic-induced inflammation
T2R38	Bacterial sensing; NO-mediated bactericidal activity	Biofilm-related infections
BBS1	19S proteasome transport; centrosome polarization	BBS (T-cell synapse defect)
BBS4	Wnt signaling regulation; B-cell maturation	BBS (Autoimmunity; B-cell defect)
BBS18	Bone marrow homeostasis; B-cell development	BBS (B-cell developmental defect)
CXCL12	Stromal niche maintenance; pre-B cell regulation	BBS (Hematopoietic abnormality)

### 3.1 Ciliary dysfunction in viral pathogenesis

In infectious diseases, the impairment of ciliary defense often constitutes a requisite step for pathogen colonization. Beyond simple mechanical dysfunction, certain viruses display distinct ciliary tropism. For instance, infection with the Delta variant of SARS-CoV-2 induces extensive deciliation and motility dysfunction in nasal epithelial cells and precipitates severe cytopathic effects (Wu et al., 2023; Tanneti et al., 2024). Similarly, influenza A virus (IAV) replicates with significantly higher efficiency in ciliated cells than in secretory cells. Single-cell transcriptomic profiling suggests that this preference is driven by the unique immunomodulatory signature of ciliated cells, characterized by a differentiated interferon-stimulated gene (ISG) profile that paradoxically creates a metabolic microenvironment conducive to viral propagation (Roach et al., 2024; Quirouette et al., 2020).

SARS-CoV-2 further exploits the ciliary architecture by anchoring its spike protein to ACE2 receptors concentrated at the ciliary distal tips (Wu et al., 2023; Lalioti et al., 2022; Lee et al., 2020; Teng et al., 2024). This high receptor density acts as an affinity trap utilizing multivalent binding to facilitate viral traversal through the periciliary layer (PCL) (Wu et al., 2023; Wang Y. et al., 2025). Notably, deciliation significantly blunts viral entry while enzymatic disruption of the overlying mucus layer accelerates it highlighting the dual role of cilia as both a protective barrier and a hijacked entry portal (Wu et al., 2023).

### 3.2 Chronic inflammation and epithelial remodeling

Ciliated cells also serve as active suppressors of allergic inflammation. Constitutive expression of IL-17D by airway cilia acts as a homeostatic brake. Conditional knockout of *Il17d* specifically in ciliated cells is sufficient to exacerbate allergic airway hyperresponsiveness and confirms these cells as the primary functional source of this protective cytokine (Yuan L. et al., 2025). In chronic conditions such as allergic rhinitis and severe asthma, the mucosal microenvironment undergoes extensive remodeling (Li Z. et al., 2025; Wang et al., 2023; Yuan et al., 2024; Boomer et al., 2024). IL-13-mediated signaling drives goblet cell metaplasia at the expense of ciliated epithelium. This transformation severely impairs mucociliary clearance and promotes pathogen persistence (Boomer et al., 2024).

In primary ciliary dyskinesia, resultant mucus stagnation drives a self-perpetuating cycle of infection and sterile inflammation (Marthin et al., 2021; Hornef et al., 2006). The inflammatory profile is genotype-specific, as shown in patients with *CCDC39* mutations who display a stronger proinflammatory cytokine signature including IL-6 and IL-8 relative to those with *DNAH5* or *DNAH11* variants (Varenyiova et al., 2023). This chronic inflammatory environment recruits neutrophils that release neutrophil elastase (Ca et al., 2025). Beyond proteolytic degradation of ciliary dynein arms to reduce ciliary beat frequency (McKelvey et al., 2021), neutrophil elastase worsens mucus dehydration by hyperactivating epithelial sodium channels and inducing CFTR endocytosis, further reinforcing the pathogenic cascade (Lee and Cohen, 2025). Therapeutic strategies targeting these pathways, such as the immunomodulatory actions of azithromycin (AZT) in supporting epithelial repair and suppressing chronic inflammation, provide promising approaches to restore cilia-associated immune homeostasis (Varenyiova et al., 2023).

### 3.3 Chemosensory dysfunction and systemic ciliopathies

The chemosensory capacity of the ciliated epithelium particularly through bitter taste receptors such as T2R38 acts as an early warning system (Lee et al., 2016; Workman et al., 2018; Hariri et al., 2017). These receptors recognize bacterial metabolites including acyl-homoserine lactones to trigger calcium-dependent nitric oxide (NO) production that enhances both bactericidal activity and ciliary motility (Cohen, 2017; Lee et al., 2014). Disruption of this chemo-immune axis increases host susceptibility to biofilm formation and refractory chronic infections (Lee and Cohen, 2025). Furthermore, environmental stressors such as nanoplastic aerosols can evade these defenses to induce the release of chemokines particularly CCL3 and drive excessive neutrophil infiltration and subsequent tissue injury (Breidenbach et al., 2025).

Finally, the connection between cilia and systemic immunity is well illustrated by Bardet-Biedl syndrome (BBS) (Cassioli et al., 2021; Tsyklauri et al., 2021; Kanie and Jackson, 2021). Clinical studies demonstrate a high prevalence of autoimmune disorders and hematopoietic abnormalities in BBS patients, suggesting that ciliary proteins are indispensable for systemic immune competence (Tsyklauri et al., 2021). Importantly, given that most hematopoietic and mature immune cells are non-ciliated in their steady state, these pathological phenotypes underscore the critical extra-ciliary roles of BBS proteins in immune regulation, independent of an axonemal structure. Mechanistically, specific BBSome components orchestrate distinct lymphoid functions. BBS1 is essential for T-cell immune synapse assembly; it couples the 19S proteasome regulatory subunit to dynein for transport to the centrosome, facilitating centrosome polarization and sustained TCR signaling (Cassioli et al., 2021). Conversely, BBS4 and BBS18 are critical for B-cell development and bone marrow homeostasis. The BBSome complex limits canonical Wnt signaling and preserves CXCL12 abundance within stromal niches, a process required for proper pre-B cell maturation (Tsyklauri et al., 2021). These observations highlight that ciliopathies are not restricted to local mucosal defects but represent systemic disorders involving both cellular and humoral immune dysfunction.

## 4 Regulatory mechanisms of ciliary immunity

The molecular and cellular regulation of ciliary-mediated immunity represents a multi-layered process that dynamically maintains host defense and physiological homeostasis.

### 4.1 Ciliary signaling, homeostasis and immune modulation

Beyond its role as a structural and mechanical barrier, metabolic and kinase-driven cascades refine ciliary signaling to modulate host defense (Picon-Galindo et al., 2022). In airway multiciliated cells, the P element-induced wimpy testes (PIWI) protein MIWI2 governs mitochondrial homeostasis and the landscape of small non-coding RNAs (Ma et al., 2009; Vasquez et al., 2025). Its deficiency leads to aberrant mitochondrial reactive oxygen species (ROS) production during influenza infection, which paradoxically accelerates viral clearance and host recovery, highlighting a complex link between ciliary metabolic states and antiviral immunity (Vasquez et al., 2025).

**FIGURE 3:**
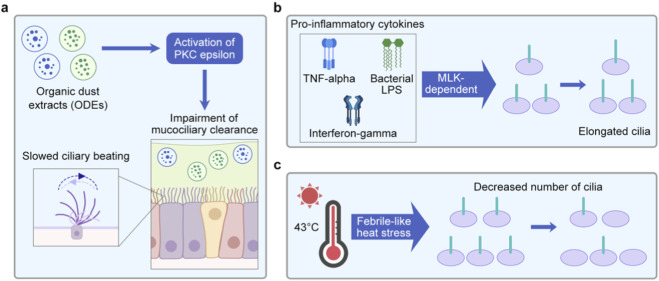
Environmental and inflammatory modulation of ciliary structure and function. **(a)** Exposure to organic dust extracts (ODEs) activates PKCε signaling, which reduces ciliary beat frequency and impairs mucociliary clearance, thereby compromising airway mucosal defense. **(b)** Pro-inflammatory mediators including TNF-α, interferon-γ, and bacterial LPS trigger MLK-dependent ciliary elongation, altering ciliary dynamics in inflammatory contexts. **(c)** Febrile-like heat stress (43°C) induces a marked reduction in ciliary incidence on epithelial cells, representing a thermal stressor that acutely disrupts ciliary homeostasis and epithelial barrier integrity.

Furthermore, the protein kinase C (PKC) and mixed lineage kinase (MLK) families act as pivotal transducers of environmental and inflammatory stimuli (Reibman et al., 2000; Wang et al., 2015; Kumari et al., 2024). Exposure to organic dust extracts (ODEs) induces ciliary slowing through the activation of PKC epsilon, which impairs axonemal dynein activity and mucociliary clearance, a process that can be pharmacologically mitigated by zinc supplementation (Bauer et al., 2024) (Figure 3a). Concurrently, ciliary architecture exhibits plastic responses to systemic inflammatory cues. Pro-inflammatory cytokines such as TNF-α, bacterial LPS and interferon-γ promote ciliary elongation via an MLK-dependent mechanism (Kumari et al., 2024) (Figure 3b). In contrast, febrile-like heat stress at 43 °C exerts an antagonistic effect by rapidly depleting the ciliated cell population without shortening existing elongated cilia (Figure 3c), illustrating that distinct inflammatory stressors utilize divergent pathways to remodel the ciliary landscape (Kumari et al., 2024).

### 4.2 Ciliary compartmentalization and secretory immunomodulation

Primary cilium functions as a dedicated signaling platform that sets activation thresholds for core inflammatory cascades. In chondrocytes and renal epithelial cells, ciliary integrity is essential for modulating NF-κB, MAPK, and JAK/STAT pathways in response to mechanical loading and pro-inflammatory cytokines (Sun et al., 2025). In nephronophthisis (NPH), loss of the basal body protein MAPKBP1 disrupts ciliary architecture and induces a switch in Jun N-terminal kinase signaling. Hyperactivated JNK promotes ciliary disassembly and impairs trafficking of phosphorylated JNK, driving chronic fibrosis and abnormal cellular proliferation (Findeisen et al., 2025). These findings indicate that ciliary dysfunction does not simply silence signaling but rewires cells toward a pro-inflammatory and dedifferentiated state that fundamentally reshapes the local cytokine secretome.

Crucially, the cilium mediates systemic immunomodulation through two distinct secretory pathways: classical cilia-dependent cytokine release and ciliary extracellular vesicle (cEV) shedding. In the immunosuppressive microenvironment of glioblastoma, the primary cilium intrinsically functions as a critical intracellular hub regulating the secretion of soluble IL-6. This cilia-dependent IL-6 release drives the recruitment of M2-type tumor-associated macrophages and the expansion of myeloid-derived suppressor cells (MDSCs) (Laws et al., 2023). Distinct from this cilia-dependent cytokine pathway, tumor cells also utilize conventional, non-ciliary EVs carrying specific microRNA signatures to globally suppress immune responses. Together, the synergy between ciliary-dependent signaling and broader EV-mediated communication promotes systemic and local immune evasion by upregulating anti-inflammatory mediators including IL-10 and TGF-β (Laws et al., 2023). Genetic ablation of ciliary components markedly reduces myeloid-derived suppressor cell infiltration and restores T-cell proliferation, establishing the primary cilium as a central regulator of the tumor-suppressive immune microenvironment.

### 4.3 Environmental sensing and adaptive ciliary remodeling

As the primary physicochemical antenna of the cell, cilia continuously integrate multimodal signals from the microenvironment to orchestrate adaptive structural and functional responses (Hilgendorf et al., 2024). However, chronic exposure to anthropogenic stressors including cigarette smoke, PM2.5 and microplastics impairs ciliary fidelity by triggering oxidative stress and inflammatory signaling cascades (Zhang et al., 2024; Bae et al., 2021; Choi et al., 2021; Theorell et al., 2026; Rangaswamy et al., 2024; Shi et al., 2024). These environmental insults drive epithelial-mesenchymal transition (EMT), compromise tight junction integrity and elicit a potent proinflammatory cytokine secretome (Feng et al., 2026). Beyond acute structural damage, emerging evidence indicates that air pollutants act synergistically with meteorological conditions and aeroallergens to exacerbate disorders such as allergic rhinitis (AR) and induce stable epigenetic alterations including aberrant DNA methylation in the nasal mucosa (Xu et al., 2025). Such remodeling suggests that environmental exposure does not only cause transient injury but may reprogram the mucosal landscape toward a state of chronic hyperreactivity.

The ciliary response to systemic physiological stress such as fever follows a distinct regulatory logic relative to local inflammatory signaling (Saba et al., 2022; Ciurkiewicz et al., 2022). Whereas proinflammatory cytokines including TNF-α, IFN-γ and LPS promote ciliary elongation via an MLK-dependent pathway, febrile-range thermal stress exerts an opposing effect. Short-term heat exposure markedly reduces ciliary prevalence without changing the length of remaining organelles, indicating that heat stress primarily impairs ciliary stability or early ciliogenesis rather than progressive resorption (Kumari et al., 2024). During respiratory infections, this thermal sensitivity may create a synergistic vulnerability in which fever amplifies direct viral cytopathy to deplete the ciliated cell pool. Such depletion severely compromises mucociliary clearance (MCC) at a critical stage when the host relies most heavily on efficient pathogen expulsion. Elucidating the signaling pathways linking environmental exposure to ciliary resorption represents a promising direction for the development of targeted antioxidants and personalized strategies to restore cilia-dependent mucosal defense.

## 5 Therapeutic potential and future directions of ciliary immunity

As the central role of cilia in immune regulation becomes increasingly appreciated, targeting cilia and their associated signaling pathways has emerged as a novel therapeutic strategy for immune-related disorders. Meanwhile, technological advances are propelling ciliary biology from descriptive structural studies toward in-depth functional analysis and precise manipulation. Nevertheless, many unresolved questions and mechanistic mysteries remain in this rapidly advancing field, warranting further investigation.

### 5.1 Precision immunomodulation via ciliary targeting

**FIGURE 4:**
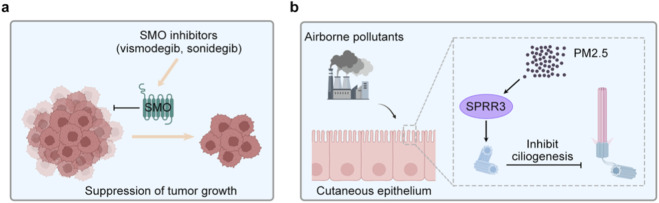
Regulation of ciliary signaling and ciliogenesis by pharmacological inhibitors and environmental pollutants. **(a)** Pharmacological inhibition of SMO by clinical small-molecule inhibitors (vismodegib and sonidegib) disrupts ciliary Hedgehog transduction, thereby suppressing tumor proliferation and growth. **(b)** Exposure to airborne particulate matter (PM2.5) upregulates SPRR3 expression in cutaneous epithelial cells, subsequently impairing physiological ciliogenesis.

The unique, segregated receptor composition of primary cilia renders them highly sensitive platforms for small-molecule intervention. In tumor immunotherapy, modulating ciliary assembly kinetics has emerged as a novel strategy to reshape the immune microenvironment. For instance, Smoothened (SMO) inhibitors such as vismodegib and sonidegib, which disrupt ciliary Hedgehog transduction, not only suppress tumor growth directly but also alter the secretion of immunosuppressive cytokines within the ciliary compartment (Otsuka et al., 2015) (Figure 4a). Given that primary cilia dynamic actions are leveraged by certain chemotherapeutics, tracking ciliary structural integrity could serve as a valuable biomarker to optimize personalized combinations of immunotherapy and microtubule-targeting agents.

Furthermore, repurposing ciliary-directed compounds represents a transformative paradigm for hereditary ciliopathies and chronic barrier inflammation. In Bardet–Biedl syndrome and related metabolic syndromes, pharmacological modulators of intraflagellar transport (such as small-molecule IFT or BBSome stabilizers) hold promise to rescue systemic immune competence and restore bone marrow hematopoietic niches (Tsyklauri et al., 2021; Kanie and Jackson, 2021; Priya et al., 2016). Within the cutaneous epithelium, where airborne pollutants (e.g., PM2.5) suppress ciliogenesis via SPRR3 upregulation (Figure 4b), topical applications targeting the SPRR3 axis or ciliary GPCRs offer novel protective avenues against atopic dermatitis and psoriasis (Bae et al., 2019; Rizaldy et al., 2021). These precise interventions highlight that the organelle can be targeted to resolve local inflammation across structurally distinct mucosal and epidermal barriers.

### 5.2 Technological frontiers in ciliary research

The continuous evolution of research methodologies remains indispensable for deepening our understanding of how ciliary structures dictate immune signaling. Recent breakthroughs in super-resolution microscopy and cryo-electron microscopy have revolutionized the visualization of ciliary architecture and mechanical signal transduction by overcoming the traditional diffraction limit (Schueder et al., 2024; Ma et al., 2025). These innovations achieve nanoscale resolution of essential ciliary substructures, including the transition zone and basal body, which serve as the primary gateways for immune-related protein trafficking (Agborbesong and Li, 2024; Djebar et al., 2024; Robinot et al., 2021). By integrating multi-omic and high-resolution imaging data, these advances bridge the gap between fundamental mechanobiology and clinical immunopathology, while providing a holistic perspective on the structural organization and functional dynamics of the organelle (Li J. et al., 2025).

At the intersection of gene editing and translational immunology, human induced pluripotent stem cell (hiPSC)-derived organoids provide high-fidelity humanized platforms (Morichon et al., 2026; Vila-Gonzal et al., 2024). Rather than merely recapitulating structural defects like renal cysts, hiPSC-derived kidney and lung organoids enable the real-time tracking of resident macrophage infiltration and cytokine profiling following ciliary ablation (e.g., NPHP1 knockout). This offers an unprecedented platform for high-throughput screening of compounds that can prevent sterile inflammation without inducing ciliotoxicity (Arai et al., 2024). Complementing these human models, specialized tissue platforms clarify the spatial logistics of mucosal tolerance. Utilizing the epididymis and intestinal loop models treated with specific Hedgehog agonists (e.g., SAG) or antagonists (e.g., cyclopamine) has unraveled how ciliary signaling orchestrates a precise transcriptomic split—selectively tempering inflammatory responses toward commensal microbes while maintaining rapid activation pathways against pathogenic challenge (Girardet et al., 2020).

### 5.3 Future horizons and unresolved enigmas in ciliary immunobiology

To translate these technological platforms and therapeutic conceptual frameworks into clinical realities, several specific mechanistic questions must first be resolved, beginning with the pronounced functional heterogeneity of cilia within the immune system. Although ciliary and cilia-associated structures are present across diverse immune cell lineages including T cells and macrophages (Picon-Galindo et al., 2022; Hua and Ferland, 2018), their lineage-specific distribution patterns and fine structural characteristics remain incompletely defined. Specifically, future research must determine whether distinct ciliary dynamic signatures can distinguish naive T cells from memory T cells, or whether ciliary architecture undergoes predictable structural remodeling during pro-inflammatory M1 versus pro-resolving M2 macrophage polarization. Elucidating these baseline cellular phenotypes will provide the essential theoretical foundation required to achieve the cell-type specificity necessary for next-generation, cilia-targeted therapeutic interventions.

Equally important is the need to clarify the molecular crosstalk between ciliary signaling and canonical immune cascades. As an integrative platform for Hedgehog and GPCR pathways, the spatial coordination of cilia with core immune receptors such as TCR and TLR remains poorly understood. Although transcriptomic data indicate that cilia-dependent signaling significantly modulates immune response genes, the precise mechanisms governing such regulation during infection or chronic inflammation require deeper investigation. Furthermore, the role of cilia in immune memory represents an underexplored field. Given that primary cilia are a hallmark of the G_0_ phase (Ho et al., 2020; Maskey et al., 2015; Jiang et al., 2020), their assembly dynamics during lymphocyte activation may fundamentally determine the threshold of secondary immune responses.

From a translational perspective, overcoming systemic toxicity and delivery barriers remains the primary bottleneck hindering the implementation of cilia-targeted therapies. Although small molecules modulating the IFT machinery or ciliary receptors hold conceptual promise, achieving tissue-specific delivery while avoiding systemic ciliotoxicity in highly ciliated organs like the kidneys and retinas represents a formidable challenge. To circumvent these adverse effects, future drug development must focus on specialized vehicles, such as antibody-drug conjugates (ADCs) or lipid nanoparticles (LNPs) functionalized with ligands for immune-specific ciliary surface markers (Wang R. et al., 2025; Wang et al., 2024; Dammes et al., 2021; Guo et al., 2026). Finally, these translational efforts must be supported by prospective cohort studies and *in vivo* mechanistic experiments to establish definitive causal relationships between environmental ciliary damage (such as that induced by PM2.5 or chronic viral infections) and long-term systemic immune dysregulation (Theorell et al., 2026). Resolving these intertwined biological and pharmacological enigmas will be essential to transition ciliary immunobiology from a burgeoning basic science into a precise clinical reality.

## 6 Conclusion

Recent advances have redefined the eukaryotic cilium as a central immunological hub that integrates mechanical barrier function and compartmentalized signal transduction to govern immune homeostasis. Motile cilia execute mucociliary clearance to establish the first line of innate mucosal defense, while primary cilia act as specialized sensory antennae that organize immune signaling cascades and maintain nuclear integrity to prevent aberrant inflammatory activation. Cilia regulate cytokine secretion and immune checkpoint presentation to coordinate innate and adaptive immune outputs, balancing antimicrobial defense, tissue repair, and pathological inflammation. Defects in ciliary structure or function triggered by infection, genetic alteration, inflammation, or environmental stress disrupt mucosal immunity and promote systemic inflammation, contributing to the pathogenesis of ciliopathies, infectious diseases, allergic disorders, and cancer immune evasion.

Ciliary immunobiology has emerged as a rapidly advancing interdisciplinary field at the interface of cell biology, immunology, and translational medicine. The compartmentalized signaling, metabolic control, and epigenetic regulation of cilia create new opportunities for targeted immunomodulation, including ciliary function repair, ciliary immune checkpoint modulation, and the repurposing of ciliotropic agents for inflammatory and neoplastic diseases. Advanced technologies enable high-resolution dissection of ciliary-immune crosstalk at cellular and subcellular scales. Future investigations will define lineage-specific functions of cilia in immune cells, identify conserved and context-dependent regulatory circuits, and overcome delivery and specificity barriers. These efforts will translate ciliary biology into next-generation therapies for immune-mediated diseases.
